# Influence of Sex-Based Differences in Cardiac Phenotype on Atrial Fibrillation Recurrence in Patients Undergoing Pulmonary Vein Isolation

**DOI:** 10.3389/fcvm.2022.894592

**Published:** 2022-07-28

**Authors:** Alena Yakimenka, Dina Labib, Steven Dykstra, Yoko Mikami, Alessandro Satriano, Jacqueline Flewitt, Patricia Feuchter, Sandra Rivest, Andrew G. Howarth, Carmen P. Lydell, F. Russell Quinn, Stephen B. Wilton, James A. White

**Affiliations:** ^1^Stephenson Cardiac Imaging Centre, Libin Cardiovascular Institute of Alberta, University of Calgary, Calgary, AB, Canada; ^2^Department of Medicine, Cumming School of Medicine, University of Calgary, Calgary, AB, Canada; ^3^Department of Cardiovascular Medicine, Cairo University, Cairo, Egypt; ^4^Department of Cardiac Sciences, Cumming School of Medicine, University of Calgary, Calgary, AB, Canada; ^5^Department of Diagnostic Imaging, Cumming School of Medicine, University of Calgary, Calgary, AB, Canada

**Keywords:** atrial fibrillation, pulmonary vein isolation, cardiovascular magnetic resonance imaging, left atrial function, gender differences, women cardiovascular health, left atrial volume, left atrial booster pump function

## Abstract

**Background:**

Pulmonary vein isolation (PVI) is a commonly engaged therapy for symptomatic atrial fibrillation (AF). Prior studies have documented elevated AF recurrence rates among females vs. males. Sex-specific mechanisms underlying this phenomenon are poorly understood. This prospective cohort study aimed to evaluate the sex-based differences in cardiac phenotype and their influence on (AF) recurrence following first-time PVI.

**Methods:**

A total of 204 consecutive patients referred for first-time PVI and 101 healthy subjects were prospectively studied by cardiovascular magnetic resonance (CMR) imaging. Multi-chamber volumetric and functional measures were assessed by sex-corrected Z-score analyses vs. healthy subjects. Patients were followed for a median of 2.6 years for the primary outcome of clinical AF recurrence. Multivariable analyses adjusting for age and comorbidities were performed to identify independent predictors of AF recurrence.

**Results:**

AF recurrence following first PVI occurred in 41% of males and 59% of females (*p* = 0.03). Females were older with higher prevalence of hypertension and thyroid disorders. Z-score-based analyses revealed significantly reduced ventricular volumes, greater left atrial (LA) volumes, and reduced LA contractility in females vs. males. Multivariable analysis revealed each of LA minimum and pre-systolic volumes and booster EF Z-scores to be independently associated with AF recurrence, providing respective hazard ratios of 1.10, 1.19, and 0.89 (*p* = 0.001, 0.03, and 0.01).

**Conclusion:**

Among patients referred for first time PVI, females were older and demonstrated significantly poorer LA contractile health vs. males, the latter independently associated with AF recurrence. Assessment of LA contractile health may therefore be of value to identify female patients at elevated risk of AF recurrence. Factors influencing female patient referral for PVI at more advanced stages of atrial disease warrant focused investigation.

## Graphical Abstract

This cohort study examined sex-based differences in cardiac phenotype and atrial fibrillation (AF) recurrence following pulmonary vein isolation (PVI). Two-hundred-four patients and 101 healthy volunteers were studied by cardiac MRI to determine z-score based measures of chamber volumes and function. Following a median 932 days of follow-up, AF recurrence occurred in 41% of males and 59% of females (*p* = 0.03). Markers of left atrial contractile health were significantly reduced in females vs. males, and were found to be independent predictors of AF recurrence.. 
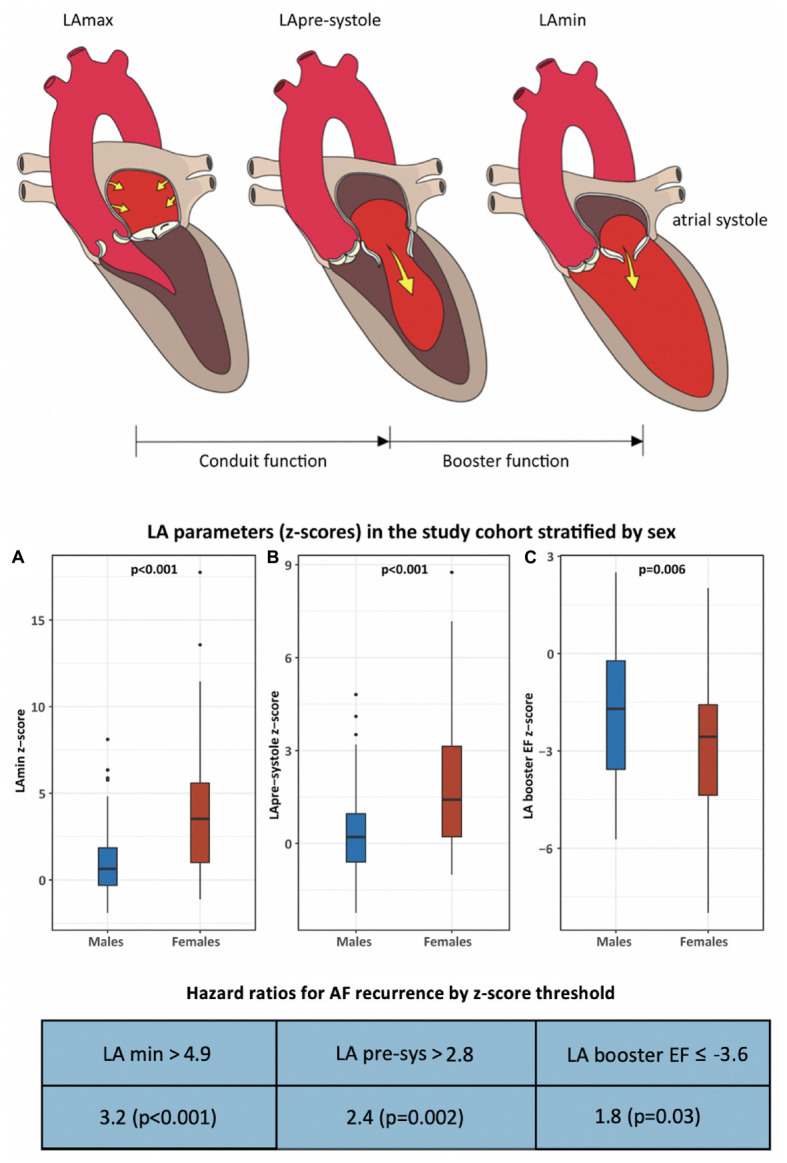

## Introduction

Atrial Fibrillation (AF) is the most common arrhythmia encountered in contemporary practice, estimated to affect over 30 million people worldwide (1). With rising prevalence over the past two decades (2), AF is recognized as an important contributor to cardiovascular hospitalization and healthcare expenditure (3). Pulmonary vein isolation (PVI) is a common invasive therapy for the treatment of symptomatic AF. Despite providing value for the improvement of symptom burden and quality of life (4, 5), AF recurrence remains common and occurs in up to 43% of patients by 1-year (6, 7), this decreasing to 20–35% at 3–5 years following the engagement of repeat interventions (8–10). Despite contributing to 43% of community reported AF, females currently represent 27% of PVI procedures (11). Among these female patients, higher rates of AF recurrence have been consistently reported vs. males (12–19). Phenotypic sex differences related to these important discrepancies have not been previously explored.

Cardiovascular magnetic resonance (CMR) imaging provides accurate and reproducible quantification of the cardiovascular phenotype in patients referred for PVI, inclusive of vascular anatomy and cardiac chamber volumetry (20). This presents unique opportunities to comprehensively study phenotypic differences between women and men that may contribute to post-PVI AF recurrence.

In this study we prospectively recruited a consecutive series of patients clinically referred for pre-procedural CMR prior to first-time PVI. We simultaneously recruited a healthy reference cohort to permit sex-matched comparisons of cardiac chamber remodeling and alterations in contractile health through use of Z-score based analysis. Respective associations for CMR-derived phenotypic markers and AF recurrence were then explored with multivariable adjustment for known confounders.

## Materials and Methods

### Study Design and Population

This was a prospective observational cohort study of patients referred for pre-procedural CMR prior to first time PVI, being a pre-defined study of the Cardiovascular Imaging Registry of Calgary (CIROC) (NCT04367220). CIROC is a prospective clinical-outcomes-based registry of the Libin Cardiovascular Institute. All patients undergo standardized capture of baseline social and clinical demographics, co-morbid illnesses, and Quality of Life (EQ-5D-3L and EQ-VAS) using a tablet-based questionnaire followed by capture of quantitative and qualitative imaging variables using commercial software (cardioDI^TM^, Cohesic Inc., Calgary, Alberta). Automated data matching of historic and prospective laboratory, pharmacy, ECG, Holter and ICD-10 coded admission and procedural data is then executed from institutional data warehouses for a period of 10 years.

Patients were recruited between March 2015 and September 2018 and followed for a median of 2.6 years for AF recurrence. Patients were excluded if they had complex congenital heart disease, severe valvular heart disease (severe stenosis or regurgitation), or prior cardiac surgery involving the atrioventricular valves. Patients were classified by AF type in accordance with contemporary Canadian Cardiovascular Society guidelines (2).

One hundred and one healthy volunteers (HV) were prospectively recruited to establish healthy reference values for non-contrast phenotypic markers. HV were recruited from the local community and required to have no history of cardiovascular disease and no moderate or severe obesity (BMI ≥ 35 kg/m^2^), hypertension, diabetes mellitus, kidney, or collagen vascular disease.

The study was approved by the Conjoint Health Research Ethics Board at University of Calgary (REB 13-0902) and all subjects provided written informed consent. All research activities were performed in accordance with the Declaration of Helsinki.

### Cardiovascular Magnetic Resonance Imaging and Analysis Protocols

CMR imaging was performed using 3 Tesla clinical scanners (Prisma or Skyra, Siemens Healthineers, Erlangen, Germany). All underwent a standardized protocol inclusive of routine cine imaging in standard 2, 3, and 4-chamber long axis views and sequential short-axis slices, 3D magnetic resonance angiography (MRA) of the pulmonary veins using a 3D gradient-echo pulse sequence followed by a volumetric interpolated breath-hold examination (VIBE). MRA was performed using a bolus of 0.2 mmol/kg Gadovist (Bayer Inc., Canada) followed by a 30cc saline flush.

Blinded analyses were performed using standardized operating procedures (SOPs) adherent to published Society of Cardiovascular Magnetic Resonance (SCMR) recommendations (20). All analyses were performed using commercially available software (cvi**42**; Circle Cardiovascular Imaging Inc., Calgary, Canada).

Ventricular volumetric analyses were performed on short axis cine images to obtain end-diastolic volume (EDV), end-systolic volume (ESV), ejection fraction (EF), and left ventricular (LV) mass. Left atrial (LA) volumes were measured at maximal (LAmax) and minimal (LAmin) volume, respectively obtained prior to atrio-ventricular valve opening and following atrial contraction using the bi-plane area-length method. LA volume was also measured prior to atrial systole (LApre-systole) for the calculation of LA booster function, a measure of active LA emptying due to atrial contraction. LA function parameters were reported as LA global, conduit, and booster EF, as previously described (21). Right atrial (RA) volumes were obtained from the 4-chamber view. All measures were indexed to body surface area (BSA) using the Mosteller formula, as appropriate. Sex-based Z-score values were calculated as the standard deviation from mean reference values of sex-matched healthy volunteers, ensuring reported differences in cardiac phenotype were independent of known sex-dependencies. Pulmonary vein and artery dimensions were measured from 3D MRA using multi-plane reconstruction (MPR) with ostial cross-sectional dimensions performed for each pulmonary vein, measured 10 mm from the atrial junction. Pulmonary artery (PA) measurements were performed for the main PA and for each branch PA at 15 mm from the main PA bifurcation.

### Pulmonary Vein Isolation Procedures

All PVI procedures were performed by percutaneous radiofrequency ablation using irrigated, contact force sensing catheters or, in a small minority, using a multielectrode pulmonary vein ablation catheter (PVAC^®^, Medtronic Inc., Minneapolis, MN, United States). Prior to ablation, 3D surface rendered models of LA anatomy were generated from CMR MRA datasets using EnsiteNavX^®^ Velocity (St Jude Medical, St. Paul, MN, United States) or Carto3^®^ system (Biosense Webster, Baldwin Park, CA, United States), providing intra-procedural guidance. The LA was catheterized under fluoroscopic guidance by femoral access followed by transseptal puncture. In all patients, the targeted procedural endpoint was complete PVI using wide antral circumferential ablation, with demonstration of bidirectional block. 3D electroanatomic mapping was performed in all cases with mapping density and intra-procedural use of this data left at the discretion of each physician. Complete isolation of the PVs was defined as elimination or dissociation of PV potentials by way of Lasso catheter. Additional LA linear ablations, most commonly a left atrial roof line, could be performed at operator discretion. Ablation of the cavotricuspid isthmus was incrementally performed for patients with a history of atrial flutter.

### Primary Clinical Outcome

The primary clinical outcome was defined as time in days to first AF recurrence following index PVI procedure. Patients were assessed at 3-months with 12-lead ECG and 24-h Holter monitoring. Clinical follow-up was subsequently performed at 6- and 12-months in out-patient clinics with 12-lead ECG and Holter monitoring ordered for patients describing palpitations. In addition, a detailed review of all 12-lead ECG’s and Holters ordered outside these visits was performed and administrative health data used to identify all emergency room or hospital visits related to AF recurrence across the Province of Alberta. The latter used ICD-10 coding from the National Ambulatory Care Reporting System (NACRS) and Discharge Abstract Database (DAD). A 1-month blanking period following PVI was applied in accordance with prior recommendations (22).

### Statistical Analysis

Continuous variables were expressed as mean ± SD or median (Q1, Q3); categorical variables as counts (percentage). We compared males and females using two-sample *t*-test/Mann-Whitney test or Chi-square/Fisher exact test for continuous and categorical variables, respectively.

Univariable and multivariable Cox proportional hazards models were constructed to investigate the relationship between predictors (demographic, procedural, and CMR variables) and AF recurrence; results were expressed as hazard ratios (HR) and 95% confidence intervals (CI). Time to event was calculated as time from first PVI procedure until first AF recurrence. Patients who did not develop recurrence were considered censored at the time of last follow-up. The assumptions of proportional hazards and linearity were verified using plots of scaled Schoenfeld and martingale residuals, respectively. We applied restricted cubic spline (RCS) transformations for variables not linearly related to the log hazard (Z-scores for LA volumes and EF), placing three pre-defined knots at the 0.05, 0.5, and 0.95 quantiles based on the number of events. HRs and 95% CI for all numerical variables were presented per one unit increase except for RCS transformed ones that were presented as partial effect plots of relative HRs against Z-scores, followed by point estimates of HRs comparing a Z-score of 2.0 vs. 1.0.

Receiver Operator Characteristic (ROC) curve analysis was used to establish an optimal univariable cut-point for Z-scores for LAmin, LApre-systole, and LA booster EF, using the maximally selected rank statistics from the “maxstat” R package (23). Kaplan-Meier curves were constructed to compare patients above and below this cut-point, with significance established by log-rank test.

Multivariable models for the overall PVI cohort were constructed to test associations of LA parameters (Z-scores) with AF recurrence, adjusting for pre-specified age, diabetes, hypertension, alcohol consumption, and pre-procedural anti-arrhythmic medication use. Z-score covariates with a *p*-value < 0.1 in univariable analysis were considered eligible for entry to multivariable models.

Analyses were conducted using R version 3.6.2, with two-tailed *p*-value < 0.05 indicating statistical significance. Survival analysis was performed using R packages “survival” version 3.1-12, “rms” version 6.0-1, and “survminer” version 0.4.9.

## Results

### Baseline Clinical Characteristics

A total of 204 patients and 101 HV were enrolled. Baseline clinical characteristics for patients, stratified by sex, are provided in Table 1; those for HV provided in Supplementary Table 1.

**TABLE 1:** Baseline non-imaging characteristics of the total study cohort, stratified by sex.

Characteristic	Overall *N* = 204	Males *N* = 153 (75%)	Females *N* = 51 (25%)	*P*-value
Age	60.2 ± 9.1	58.9 ± 8.9	64.2 ± 8.7	<0.001*
Body surface area (BSA, m^2^)	2.1 ± 0.2	2.2 ± 0.2	1.9 ± 0.2	<0.001*
Body mass index (BMI, kg/m^2^)	28.8 ± 4.8	28.9 ± 4.3	28.4 ± 5.9	0.60
Obesity (BMI ≥30 kg/m^2^)	66 (32%)	51 (33%)	15 (29%)	0.60
Heart rate, bpm	58.0 (52.0, 70.0)	57.0 (51.0, 68.2)	60.0 (55.5, 72.0)	0.039*
Systolic BP, mmHg	115.9 ± 14.0	115.3 ± 12.7	117.8 ± 17.3	0.6
Diastolic BP, mmHg	70.0 ± 9.7	70.5 ± 9.3	68.5 ± 10.8	0.24
Dyspnea (NYHA class II-IV)	66 (34%)	46 (32%)	20 (43%)	0.17
Diabetes mellitus^†^	10 (4.9%)	7 (4.6%)	3 (5.9%)	0.71
Hypertension	56 (27%)	36 (24%)	20 (39%)	0.030*
Hyperlipidemia^‡^	141 (69%)	105 (69%)	36 (71%)	0.79
Hypothyroidism	27 (13%)	11 (7%)	16 (31%)	<0.001*
Hyperthyroidism	5 (2%)	1 (1%)	4 (8%)	0.014*
CKD^§^	18 (9%)	6 (4%)	12 (24%)	<0.001*
Smoking				0.96
Never	150 (78%)	112 (77%)	38 (81%)	
Current	22 (11%)	17 (12%)	5 (11%)	
Former	20 (10%)	16 (11%)	4 (9%)	
Alcohol consumption				0.67
None	24 (12%)	17 (11%)	7 (14%)	
Occasional (<1 drink/day)	139 (68%)	103 (68%)	36 (71%)	
Regular (at least 1 drink/day)	40 (20%)	32 (21%)	8 (16%)	
Caffeine consumption				0.009*
None	9 (4.7%)	3 (2.1%)	6 (13%)	
Occasional (<1 drink/day)	59 (31%)	43 (29%)	16 (34%)	
Regular (at least 1 drink/day)	125 (65%)	100 (68%)	25 (53%)	
QoL (rating on 0–100 scale)	80.0 (70.0, 85.0)	80.0 (70.0, 85.0)	77.5 (70.0, 85.0)	0.79
Medications				
Aspirin	25 (12%)	21 (14%)	4 (8%)	0.27
Beta-blocker	136 (67%)	100 (65%)	36 (71%)	0.49
ACEi/ARB	64 (31%)	47 (31%)	17 (33%)	0.73
Calcium channel blocker	46 (23%)	33 (22%)	13 (25%)	0.56
Anti-coagulant	190 (93%)	142 (93%)	48 (94%)	0.999
Anti-arrhythmic	145 (71%)	112 (73%)	33 (65%)	0.25
Digoxin	12 (6%)	9 (6%)	3 (6%)	0.999
Loop diuretic	22 (11%)	12 (7.8%)	10 (20%)	0.019*
Lipid lowering	67 (33%)	52 (34%)	15 (29%)	0.55
Atrial fibrillation type Paroxysmal (vs. persistent)	132 (65%)	98 (64%)	34 (67%)	0.74
Labs				
Hemoglobin, g/L	148.6 ± 12.6	151.9 ± 11.0	138.6 ± 11.6	<0.001*
GFR, ml/min/1.73 m^2^	89.3 (73.4, 105.7)	94.9 (77.3, 113.9)	77.5 (61.0, 94.1)	<0.001*

*Values are mean ± SD, median (IQR), or number (%). NYHA indicates New York Heart Association; CKD, chronic kidney disease; QoL, quality of life; ACEi, angiotensin converting enzyme inhibitor; ARB, angiotensin receptor blocker; BMI, body mass index; and GFR, glomerular filtration rate. Bold values indicate p < 0.05.*

*p < 0.05.

*^†^Coded as present if the patient was receiving oral hypoglycemics or insulin or had a HbA1C ≥ 6.5% within 3 years prior to or 4 months following index CMR.*

*^‡^Coded as present if the patient was receiving lipid-lowering therapy or had an LDL-C ≥ 3.5 mmol/L or triglycerides ≥ 1.7 mmol/L within 3 years prior to or 4 months following index CMR.*

*^§^Defined as GFR < 60 ml/kg/m^2^.*

The mean age of patients was 60.2 ± 9.1 years (range 32–80 years), with 25% being female. A total of 132 (65%) patients had paroxysmal AF with the remaining having persistent AF. Forty-two patients (21%) had a concurrent history of atrial flutter. As shown in Table 1, female patients were significantly older (mean difference 5.3 years), with a higher proportion having hypertension (39 vs. 24%, *p* = 0.03), hypothyroidism (31 vs. 7%, *p* < 0.001), or hyperthyroidism (8 vs. 1%, *p* = 0.01) vs. males. A similar prevalence of diabetes, hyperlipidemia, and obesity was seen. Patient survey responses yielded lower rates of regular (≥ 1 per day) caffeine consumption among females (53 vs. 68%, *p* = 0.009) but similar smoking and alcohol intake.

Baseline medication use was similar between sexes except for increased loop diuretic use in females (20 vs. 8%; *p* = 0.02). Anti-arrhythmic therapy was being used at time of CMR imaging in 71% of subjects, with no difference between sexes. Compared to males, females had a lower mean GFR and higher overall prevalence of chronic kidney disease, defined as GFR < 60 ml/kg/m^2^ (24 vs. 4%, *p* < 0.001).

### Pulmonary Vein Isolation Procedural Details

Median duration from CMR to first PVI was 24.5 (8.3, 53.0) days with no differences between sexes. Table 2 summarizes all relevant intra-procedural variables. Complete electrical pulmonary vein isolation was achieved in 100% of females and 93% of males. There were no observed differences between sexes in any procedural variable.

**TABLE 2:** Catheter ablation procedure details for the overall cohort and stratified by sex.

Characteristic	Overall *N* = 204	Males *N* = 153 (75%)	Females *N* = 51 (25%)	*p*-value
Sinus rhythm at onset of procedure	123 (61%)	90 (59%)	33 (67%)	0.31
Ablation count	55.0 (44.0, 75.0)	55.5 (44.2, 74.8)	55.0 (39.0, 75.0)	0.341
Total ablation time, s	2809.0 (2291.5, 3562.5)	2864.0 (2343.0, 3618.0)	2713.0 (2198.5, 3294.5)	0.11
Maximum power, watts	30.0 (25.0, 31.0)	30.0 (25.0, 31.0)	30.0 (25.0, 31.0)	0.73
Complete electrical PVI achieved	194 (95%)	143 (93%)	51 (100%)	0.069
Concurrent Atrial flutter ablation	37 (18%)	25 (16%)	12 (24%)	0.25
LA roof line	12 (5.9%)	6 (3.9%)	6 (12%)	0.077
Posterior isolation/posterior box	3 (1.5%)	3 (2.0%)	0 (0%)	0.57
PVAC catheter used	4 (2.0%)	3 (2.0%)	1 (2.0%)	0.999
3D mapping system used	202 (99%)	151 (99%)	51 (100%)	0.999

*Values are mean ± SD, median (IQR), or number (%).*

*AF indicates atrial fibrillation; PVI, pulmonary vein isolation; and LA, left atrium.*

### Sex-Based Differences in Cardiovascular MRI Phenotype

All patients completed CMR imaging. Despite active rhythm control strategies, AF was present at time of CMR in 25 (12%) patients, preventing quantification of atrial function in these subjects. A single male patient had mild to moderate aortic and mitral regurgitation.

Baseline chamber volumes, mass, and EF stratified by sex, are shown in Table 3, and displayed according to both non-Z-score and Z-score-based values. Non-Z-score values showed females to have smaller BSA-indexed LV volumes and mass, similar LV EF, smaller RV volumes, and relatively higher RV EF (58.0 vs. 52.6%, *p* < 0.001) vs. males. BSA-indexed LAmax volume was similar between sexes. Of 179 patients in sinus rhythm at time of CMR, BSA-indexed LApre-systole and LAmin measurements were 11 and 26% higher in females, respectively (*p* = 0.02 for each). LA function was significantly lower in females by both LA global and conduit EF measures. Z-score correction demonstrated a further reduction in all LA function parameters in women vs. men when compared to sex-matched controls, with LA booster EF becoming highly significant (*p* = 0.006).

**TABLE 3:** Baseline CMR chamber volumes and ejection fraction of the overall cohort and stratified by sex.

CMR variables	Overall *N* = 204	Males *N* = 153 (75%)	Females *N* = 51 (25%)	*p*-value
**Non-Z-score values**				
LV EDV, ml/m^2^	78.9 ± 15.1	82.1 ± 14.5	69.6 ± 12.8	<0.001*
LVESV, ml/m^2^	32.0 ± 10.2	33.8 ± 10.9	26.9 ± 5.1	<0.001*
LVEF,%	59.2 ± 9.0	58.8 ± 9.3	60.6 ± 7.9	0.18
LV mass, g/m^2^	52.8 ± 11.5	55.8 ± 10.5	44.0 ± 9.8	<0.001*
RVEDV, ml/m^2^	85.5 ± 18.9	90.5 ± 17.3	70.7 ± 15.6	<0.001*
RVESV, ml/m^2^	39.4 ± 11.1	42.7 ± 9.9	29.7 ± 8.4	<0.001*
RVEF,%	53.9 ± 7.9	52.6 ± 7.6	58.0 ± 7.4	<0.001*
LAmax, ml	83.0 (67.9, 102.7)	84.4 (69.5, 105.0)	73.8 (65.6, 97.0)	0.17
LAmax, ml/m^2^	39.4 (32.4, 48.0)	39.4 (30.5, 46.7)	39.3 (34.7, 51.4)	0.094
LAmin, ml	41.6 (29.7, 58.5)	41.6 (29.7, 58.6)	41.7 (29.7, 55.1)	0.75
LAmin, ml/m^2^	20.1 (14.5, 27.0)	18.9 (13.6, 25.7)	23.9 (16.3, 30.1)	0.023*
LApre-systole, ml	60.3 (47.0, 77.3)	62.8 (47.0, 77.0)	60.0 (47.3, 77.5)	0.80
LApre-systole, ml/m^2^	29.3 (22.5, 35.8)	27.9 (21.3, 34.0)	31.1 (24.5, 40.5)	0.017*
LA global EF,%	48.1 (36.2, 57.5)	51.0 (37.2, 59.1)	43.5 (32.9, 54.2)	0.017*
LA booster EF,%	29.7 (17.7, 39.8)	29.7 (16.0, 40.7)	28.9 (19.3, 34.2)	0.40
LA conduit EF,%	24.9 (17.2, 31.7)	26.1 (18.6, 33.3)	23.6 (13.1, 29.0)	0.018*
**Z-score values by sex†**				
LV EDV	–0.4 ± 1.2	–0.2 ± 1.2	–0.7 ± 1.1	0.011*
LV ESV	0.2 ± 1.6	0.4 ± 1.8	–0.1 ± 0.8	0.012*
LV EF	–1.1 ± 2.3	–1.1 ± 2.5	–0.8 ± 1.6	0.35
LV mass	–0.1 (–0.8, 0.5)	–0.2 (–0.7, 0.4)	0.1 (–0.9, 1.3)	0.17
RV EDV	–0.6 ± 1.3	–0.4 ± 1.3	–1.0 ± 1.2	0.009*
RV ESV	–0.2 ± 1.2	0.0 ± 1.3	–0.6 ± 1.1	0.002*
RV EF	–0.6 ± 1.7	–0.7 ± 1.7	–0.2 ± 1.5	0.061
LAmax	0.3 (–0.6, 1.3)	0.2 (–0.8, 0.9)	0.5 (–0.2, 2.2)	0.003*
LAmin	1.1 (–0.1, 2.7)	0.6 (–0.3, 1.9)	3.5 (1.0, 5.6)	<0.001*
LApre-systole	0.5 (–0.4, 1.5)	0.2 (–0.6, 1.0)	1.4 (0.2, 3.1)	<0.001*
LAglobal EF	–1.9 (–3.7, –0.5)	–1.3 (–3.3, –0.2)	–3.2 (–4.9, –1.4)	<0.001*
LAbooster EF	–2.0 (–3.9, –0.4)	–1.7 (–3.6, –0.2)	–2.6 (–4.4, –1.6)	0.006*
LA conduit EF	–0.8 (–1.6, 0.0)	–0.5 (–1.3, 0.2)	–1.2 (–2.2, –0.6)	<0.001*

*Values are mean ± SD or median (IQR). LV indicates left ventricle; EDV, end-diastolic volume; ESV, end-systolic volume; EF, ejection fraction; RV, right ventricle; LA, left atrium; LAmax, maximum LA volume; LAmin, minimum LA volume; and LApre-systole, LA volume pre-atrial systole. Bold values indicate p < 0.05.*

*p < 0.05.

*†Z-score values calculated from sex-stratified reference values (BSA-adjusted for all volume and mass measures) from a healthy reference cohort (n = 101).*

Observed differences in RA volumes, pulmonary venous and arterial measurements are provided in Supplementary Table 2. BSA-indexed RAmax and RAmin volumes were smaller in females vs. males (*p* = 0.02 and *p* = 0.008, respectively). 3D-MRA derived cross-sectional areas of pulmonary vein ostia were similar between sexes except for the right superior pulmonary vein which was smaller in females (*p* = 0.03). A similar prevalence of a left common trunk and separate right middle pulmonary vein was observed between sexes. No differences in main or left PA cross-sectional areas were identified, while females had modestly smaller right PAs (*p* = 0.02).

### Influence of Baseline Clinical Characteristics on Atrial Fibrillation Recurrence

Overall, 93 patients (46%) met criteria for AF recurrence over a median follow up period of 932 days (Q1-Q3 671–1,300). The 1-year cumulative incidence rate of AF recurrence was 29%. AF recurrence over the study period was significantly more common in females (59%) vs. males (41%, *p* = 0.03). Of the 93 patients with AF recurrence, 60 (16 females and 44 males) subsequently underwent a repeat PVI procedure. A single patient was lost to follow-up at 245 days with no documented AF recurrence, and another died at 63 days without documented AF recurrence and no cause of death being identified. Both were classified as negative with respect to AF recurrence.

**FIGURE 1:**
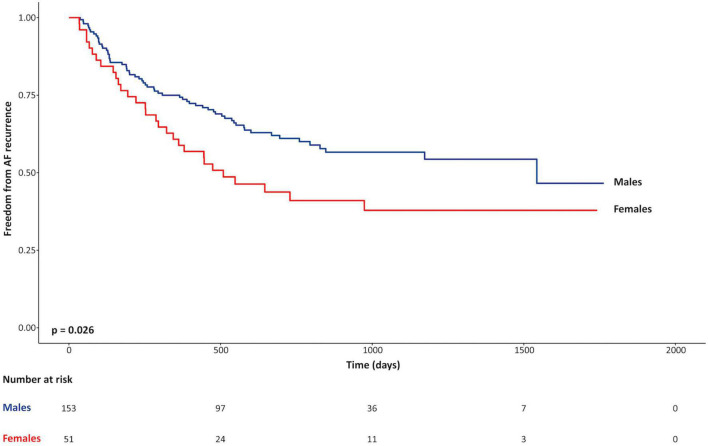
Kaplan-Meier curves for freedom from recurrence of atrial fibrillation for females vs. males.

Univariable associations of non-imaging parameters with the primary clinical outcome are shown in the Table 4. Female sex was the only clinical variable positively associated with the primary outcome (unadjusted HR 1.6, *p* = 0.03). Kaplan-Meier curves, shown in Figure 1, illustrate the influence of female sex on freedom from AF recurrence in the study population. Use of pre-procedural anti-arrhythmic medications at the time of CMR was the only clinical variable showing a significant protective association with AF recurrence (HR 0.6; *p* = 0.02). Regular alcohol consumption showed a protective trend (HR 0.6; *p* = 0.06). Of note, among regular alcohol drinkers, only 3 male patients described heavy drinking (≥ 3 drinks/day); all remaining patients reporting an intake of 1–2 drinks/day.

**TABLE 4:** Univariable associations of non-imaging variables with the primary clinical outcome in the overall cohort (both sexes).

Characteristic	HR (95% CI)	*p*-value
Age, per 1 year	0.9995 (0.977–1.023)	0.963
Female sex	**1.633 (1.057–2.524)**	0.027*
Dyspnea (NYHA class II-IV)	1.149 (0.748–1.764)	0.53
Diabetes mellitus	0.545 (0.173–1.724)	0.30
Hypertension	1.085 (0.693–1.698)	0.72
Hyperlipidemia	0.802 (0.523–1.230)	0.31
Hypothyroidism	1.475 (0.860–2.531)	0.16
Hyperthyroidism	0.733 (0.181–2.978)	0.66
Chronic kidney disease	1.443 (0.748–2.784)	0.27
Smoking		
Never	Reference category	
Current	1.003 (0.516–1.950)	0.99
Former	1.054 (0.542–2.049)	0.88
Regular alcohol consumption (at least 1 drink/day)^†^	0.575 (0.320–1.033)	0.064
Caffeine consumption		
None	Reference category	
Occasional (< 1 drink/day)	0.660 (0.270–1.610)	0.36
Regular (at least 1 drink/day)	0.676 (0.290–1.576)	0.37
QoL (rating on 0–100 scale)	0.998 (0.986–1.011)	0.80
Baseline medications		
Aspirin	1.139 (0.633–2.047)	0.66
Beta-blocker	1.058 (0.685–1.634)	0.80
ACEi/ARB	1.07 (0.695–1.647)	0.76
Calcium channel blocker	1.290 (0.810–2.054)	0.28
Anti-coagulant	1.147 (0.501–2.628)	0.75
Anti-arrhythmic	**0.602 (0.394–0.919)**	0.019*
Digoxin	0.451 (0.142–1.427)	0.18
Loop diuretic	1.422 (0.791–2.556)	0.24
Lipid-lowering	0.746 (0.476–1.167)	0.20
Body surface area, per m^2^	0.662 (0.275–1.596)	0.36
BMI, per 1 kg/m^2^	0.989 (0.947–1.033)	0.63
Obesity (BMI ≥30 kg/m^2^)	0.977 (0.633–1.510)	0.92
Heart rate, per 1 bpm	1.008 (0.999–1.018)	0.095
Systolic blood pressure, per 1 mmHg	1.013 (0.998–1.028)	0.098
Diastolic blood pressure, per 1 mmHg	1.005 (0.983–1.027)	0.68
Atrial fibrillation type—non-paroxysmal (vs. paroxysmal)	1.300 (0.856–1.973)	0.22
Labs		
Hemoglobin, per 1 g/L	0.993 (0.978–1.009)	0.38
GFR, per 1 ml/min/1.73 m^2^	1.001 (0.993–1.008)	0.87
Ablation procedure		
Sinus rhythm at onset of procedure	0.755 (0.499–1.144)	0.18
Ablation count	0.998 (0.990–1.006)	0.66
Total ablation time, per 1 s	0.999 (0.999–0.999)	0.54
Maximum power, per 1 watt	0.990 (0.924–1.061)	0.78
Left atrial roof line	1.983 (0.960–4.099)	0.065
Posterior isolation/posterior box	1.525 (0.375–6.199)	0.56
PVAC catheter used	1.007 (0.248–4.091)	0.99
3D mapping system used	1.054 (0.147–7.566)	0.96
Complete pulmonary vein isolation achieved	0.875 (0.355–2.157)	0.77

*HR indicates hazard ratio; CI, confidence interval; NYHA, New York Heart Association; CKD, chronic kidney disease; QoL, quality of life; ACEi, angiotensin converting enzyme inhibitor; ARB, angiotensin receptor blocker; BMI, body mass index; and GFR, glomerular filtration rate. Bold values indicate p < 0.05.*

*p < 0.05.

*^†^Among regular drinkers, only 3 male patients were heavy drinkers (3–4 drinks/day), all remaining patients reported 1–2 drinks/day.*

### Influence of Sex-Corrected Phenotype Markers on Atrial Fibrillation Recurrence

**FIGURE 2:**
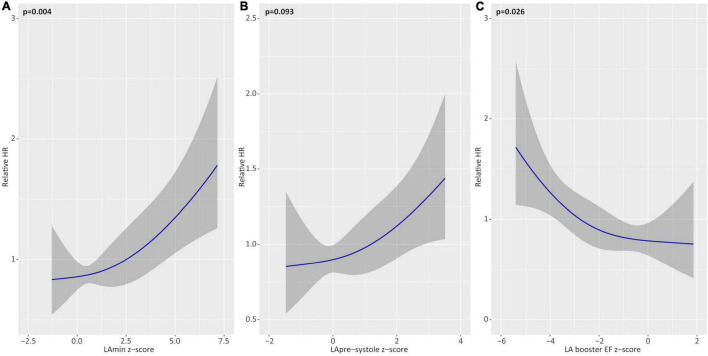
Univariable associations of left atrial (LA) parameters with the primary outcome. Unadjusted hazards of atrial fibrillation recurrence for Z-score based deviations (relative to healthy reference values) for minimum LA volume (LAmin; **A**), pre-systole LA volume (LApre-systole; **B**), and LA booster ejection fraction (EF; **C**). Relative hazard ratios plotted across the range of restricted cubic spline-transformed variables.

Univariable associations for Z-score reported (corrected for sex-specific reference values) chamber volumes, EF, and LV mass with the primary outcome are shown in Table 5 and Figure 2. Z-score measures for LA booster EF were inversely associated with the primary outcome (HR 0.98 for a Z-score of 2.0 vs. 1.0; *p* = 0.03). LAmin was similarly associated with the primary outcome, demonstrating a HR of 1.07 for a Z-score of 2.0 vs. 1.0 (*p* = 0.004), where-as LApre-systole volume showed a trend with HR 1.15 (*p* = 0.09). RA volumes, pulmonary venous, and PA ostial dimensions were not associated with the primary outcome (Supplementary Table 3).

**TABLE 5:** Univariable associations of Z-score values for CMR chamber volumes and ejection fraction with the primary clinical outcome in the overall cohort (both sexes).

CMR variables	HR (95% CI)*	*p*-value
LV EDV	1.115 (0.939–1.325)	0.21
LV ESV	1.038 (0.913–1.180)	0.57
LV EF	1.020 (0.932–1.117)	0.66
LV mass	1.138 (0.945–1.371)	0.17
RV EDV	1.079 (0.925–1.259)	0.34
RV ESV	1.046 (0.886–1.236)	0.59
RV EF	1.033 (0.912–1.170)	0.61
LAmax**^†^**	1.026 (0.859–1.225)	0.54
LAmin**^†^**	**1.073 (0.953–1.208)**	**0.004^‡^**
LApre-systole**^†^**	1.147 (1.014–1.298)	0.093
LA global EF**^†^**	0.916 (0.833–1.006)	0.19
LA booster EF**^†^**	**0.979 (0.763–1.256)**	**0.026^‡^**
LA conduit EF**^†^**	0.899 (0.692–1.167)	0.89

*HR indicates hazard ratio; CI, confidence interval; LV, left ventricle; EDV, end-diastolic volume; ESV, end-systolic volume; EF, ejection fraction; RV, right ventricle; LA, left atrium; LAmax, maximum LA volume; LAmin, minimum LA volume; and LApre-systole, LA volume pre-atrial systole. Bold values indicate p < 0.05.*

*Calculated per 1 unit increase in Z-score for all variables, except for restricted cubic spline (RCS) transformed ones calculated for a Z-score of 2.0 vs. 1.0.

***^†^**RCS transformation used, with HR and 95% CI calculated for a Z-score of 2.0 vs. 1.0.*

***^‡^**p < 0.05.*

**FIGURE 3:**
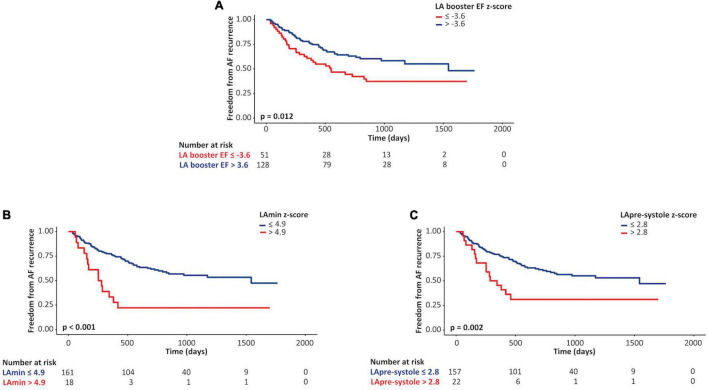
Kaplan-Meier curves for left atrial (LA) parameters as predictors of AF recurrence. Unadjusted Kaplan-Meier curves describing associations between lower vs. higher Z-scores of LA booster ejection fraction (EF; **A**), minimum LA volume (LAmin; **B**), and pre-systole LA volume (LApre-systole; **C**) values and freedom from atrial fibrillation recurrence following pulmonary vein ablation.

ROC curve analysis was performed to identify optimal Z-score thresholds for LAmin, LApre-systole, and LA booster EF for prediction of AF recurrence. Patients with a Z-score booster EF less than or equal to a threshold of –3.6 experienced an 1.76-fold risk of AF recurrence (*p* = 0.03); those with Z-score LAmin or LApre-systole above 4.9 and 2.8 experienced respective 3.22-fold (*p* < 0.001) and 2.4-fold (*p* = 0.002) increased risk of AF recurrence, as illustrated in Figure 3.

**FIGURE 4:**
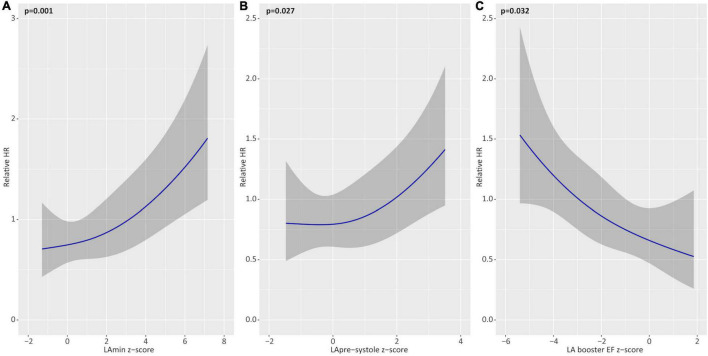
Multivariable associations of left atrial (LA) parameters with the primary outcome. Adjusted hazards of atrial fibrillation recurrence for Z-score based deviations (relative to healthy reference values) for minimum LA volume (LAmin; **A**), pre-systole LA volume (LApre-systole; **B**), and LA booster ejection fraction (EF; **C**). Shown as partial effect plots for multivariable associations with relative hazard ratios plotted across the range of restricted cubic spline-transformed variables. All models are adjusted for age, hypertension, diabetes, alcohol consumption, and pre-procedural anti-arrhythmic medication use.

Multivariable models were constructed to identify independent associations between Z-score (sex-corrected) phenotype markers and future AF recurrence. Separate models were constructed assessing the influence of LA booster EF, LAmin, and LApre-systole on the primary outcome, adjusted for age, diabetes, hypertension, regular alcohol consumption, and pre-procedural anti-arrhythmic medication use (Table 6). In these models, LA booster EF was found to be independently protective for the primary outcome, providing a HR 0.89 for a Z-score of 2.0 vs. 1.0 (*p* = 0.01). Similarly, Z-score LAmin and LApre-systole were independently associated with the primary outcome with respective HR of 1.10 and 1.19 for Z-scores of 2.0 vs. 1.0 (respective *p* = 0.001 and 0.03). In all models, regular alcohol consumption and anti-arrhythmic medication use remained independently protective from the primary outcome. Figure 4 provides adjusted relative hazard ratios for each of the studied LA parameters according to sex-based Z-scores. Similar results were obtained on repeat multivariable analysis adjusting separately for each of hyperthyroidism and hypothyroidism, in addition to age, diabetes, hypertension, regular alcohol consumption, and pre-procedural anti-arrhythmic medication use.

**TABLE 6:** Multivariable models for associations of Z-scores for left atrial CMR parameters with the primary clinical outcome in overall cohort (both sexes).

Parameter	HR (95% CI)*	*p*-value
**Model 1. LAmin**		
Age, per 1 year	0.967 (0.966–1.242)	0.45
Diabetes	0.525 (0.158–1.742)	0.29
Hypertension	1.341 (0.809–2.223)	0.25
Regular alcohol consumption (at least 1 drink/day)^†^	**0.498 (0.262–0.949)**	**0.034^‡^**
Pre-procedural anti-arrhythmic medication	**0.500 (0.312–0.801)**	**0.004^‡^**
LAmin^§^	**1.096 (0.967–1.242)**	**0.001^‡^**
**Model 2. LApre-systole**		
Age, per 1 year	0.993 (0.968–1.019)	0.60
Diabetes	0.575 (0.175–1.891)	0.36
Hypertension	1.309 (0.793–2.159)	0.29
Regular alcohol consumption (at least 1 drink/day)^†^	**0.513 (0.270–0.973)**	**0.041^‡^**
Pre-procedural anti-arrhythmic medication	**0.511 (0.314–0.829)**	**0.007^‡^**
LApre-systole^§^	**1.185 (1.046–1.342)**	**0.027^‡^**
**Model 3. LA booster EF**		
Age, per 1 year	1.001 (0.977–1.026)	0.92
Diabetes	0.580 (0.176–1.912)	0.37
Hypertension	1.428 (0.848–2.403)	0.18
Regular alcohol consumption (at least 1 drink/day)^†^	**0.495 (0.260–0.940)**	**0.032^‡^**
Pre-procedural anti-arrhythmic medication	**0.498 (0.311–0.798)**	**0.004^‡^**
LA booster EF^§^	**0.885 (0.681–1.152)**	**0.013^‡^**

*HR indicates hazard ratio; CI, confidence interval; LA, left atrium; LAmin, minimum LA volume; and LApre-systole, LA volume pre-atrial systole; LApre-systole, LA volume pre-atrial systole; and EF, ejection fraction. Bold values indicate p < 0.05.*

*Calculated per 1 unit increase for all numerical variables, except for restricted cubic spline (RCS) transformed ones calculated for a Z-score of 2.0 vs. 1.0.

*^†^Among regular drinkers, only 3 male patients were heavy drinkers (3–4 drinks/day), all remaining patients reported 1–2 drinks/day.*

*^‡^p < 0.05.*

*^§^RCS transformation used, with HR and 95% CI calculated for a Z-score of 2.0 vs. 1.0.*

### Sensitivity Analysis

Of 93 total AF recurrence events, all but 6 were based on documented AF using an available ECG or Holter recording, these available for all local hospitals. The incremental use of ICD-10 coded AF hospitalization across the Province of Alberta, this used to identify events beyond the local ECG data repository, yielded an additional 6 events. Sensitivity analysis removing these administratively only coded events did not alter the results of analysis, as detailed in Supplementary Tables 4–6 and Supplementary Figures 1, 2.

## Discussion

This is the first prospective study dedicated to identifying sex-based differences in cardiac phenotype and their related influence on AF recurrence in patients undergoing first time PVI. Our study identified an 18% absolute increase in the incidence of AF recurrence in females vs. males during the study period, this strongly associated with reductions in left atrial contractile health. Versus male patients, females showed significantly higher LAmin and LApre-systole volumes and lower LA booster function relative to sex-matched reference values, these markers being independently associated with AF recurrence following multivariable adjustment. These observations provide unique insights into sex-related differences among patients referred for PVI, and offer support for a greater severity of atrial myopathy that contributes to the higher observed incidence of AF recurrence in females.

Elevated rates of AF recurrence in females following PVI have been reliably observed in numerous studies (12–19). Over a decade ago, a landmark study by Patel et al. demonstrated that, among 3,265 consecutively studied patients undergoing PVI, women experienced significantly lower freedom from AF recurrence compared to men (68.5 vs. 77.5% < 0.001) over a median follow-up of 24 months (14). Following this, a subgroup analysis of the Fire and Ice trial showed female gender to be associated with a 37% increased risk in AF recurrence (HR 1.37; 95% CI, 1.08–1.73; *p* = 0.010) (24), findings that have since been replicated in CABANA trial (25).

Our current study provides strong support for greater relative reductions in LA contractile health in females vs. males at time of referral to PVI. While no prior study has focused on identifying sex-based differences in cardiac phenotype in this referral population, one prior study by Yu et al., identified surrogate evidence of reduced LA contractile health in females using TEE-based LA appendage Doppler interrogation. In this study females experienced a significantly higher rate of AF recurrence vs. males (39 vs. 27%, *p* < 0.001) and demonstrated significantly lower LA appendage emptying velocities (13).

Without targeted focus on sex phenotypic differences, several prior echocardiography studies have identified population level associations between LA contractile function and AF recurrence (26–31). Similar studies leveraging CMR markers of LA contractile health have also shown population wide associations. Several CMR-based studies have replicated these findings, using both volumetric and strain-based analyses (32–35). Collectively, these studies have provided a foundation for establishing LA health, as assessed by contractile performance, to be of central importance for the maintenance of sinus rhythm following PVI. Our current study expands on these observations by identifying important sex-related differences in atrial contractile health, identifying females to have a greater relative reduction in these markers at time of PVI referral vs. males, and that this is associated with elevation in rates of AF recurrence.

While associations between atrial contractile performance and fibrosis burden by MRI have shown poor correlation (36), reductions in atrial contractility are anticipated to accompany the adverse atrial remodeling (i.e., fibrosis) observed in chronic AF populations (37–39), the latter is strongly associated with AF recurrence (36, 39). However, to our knowledge, no study has examined sex-related differences in atrial remodeling or contractile function in AF referral populations. Both animal models (40) and population MRI-based investigations (41) have, however, suggested sex-related differences in remodeling that occurs at the ventricular level.

## Limitations

As a single center study with potential for referral bias, our study findings would benefit from external validation. We observed that females in our referral cohort showed a higher mean age than males, this potentially reflecting referral bias toward later stage referral of females to PVI procedures in clinical practice. Z-scores for the LA metrics were stratified for sex but not for age. Despite efforts to recruit healthy volunteers of similar age, challenges were experienced in identifying qualifying healthy subjects aged > 60 years. However, observed sex differences in event rates were maintained following adjustment for age. We also ensured that the predictive utility of LAmin, LApre-systole, and booster EF, demonstrated to be higher among females, was maintained following adjustment for age and all other relevant covariates. As with all clinical observational studies evaluating AF recurrence, lack of continuous ambulatory surveillance limits the capture of asymptomatic or brief AF episodes. Accordingly, such episodes may be missed, providing a potential limitation to the study design. We also acknowledge the inability to execute a time-dependency analysis of the influence of anti-arrhythmic medication prescription at different time-points following ablation. Finally, while of interest, our CMR imaging protocol was not designed to directly evaluate measures of LA fibrosis by advanced 3D acquisition techniques, and therefore correlation to this marker was not permitted.

## Conclusion

Female patients experience higher rates of AF recurrence following first time PVI for the treatment of paroxysmal or persistent AF. Using pre-procedural CMR-based phenotyping with z-score correction to sex-matched controls, we observed females to have significantly greater reductions in LA contractile health at time of PVI referral compared to males. This finding was strongly associated with future AF recurrence. Our findings support that female patients have more advanced atrial myopathy at time of referral to PVI, providing pathophysiologic substrate for the higher observed rate of AF recurrence in this population. Efforts to improve access to PVI for female patients at earlier stages of AF care may be of importance to improve procedural outcomes in this population.

## Data Availability Statement

The raw data supporting the conclusions of this article will be made available from the corresponding author, on reasonable request.

## Ethics Statement

The studies involving human participants were reviewed and approved by the Conjoint Health Research Ethics Board at University of Calgary (REB 13-0902). The patients/participants provided their written informed consent to participate in this study.

## Author Contributions

AY performed patient data collection, image analysis, and manuscript authorship. JW was senior author and conceived, designed, edited, and finalized manuscript content. DL performed statistical analysis and manuscript revision. SD, YM, AS, and JF structured related data collection, data analysis, and manuscript review. PF performed image acquisition. SR performed patient recruitment and data collection. AH, CL, FQ, and SW participated in patient recruitment and manuscript revision. All authors contributed to the article and approved the submitted version.

## Conflict of Interest

JW received funding from the Canadian Institute of Health Research (CIHR), received research support from Siemens Healthineers, and was a shareholder of Cohesic Inc. AH received consulting fees from Amgen and was a shareholder of Cohesic Inc. JF was a shareholder of Cohesic Inc. SW received funding from CIHR, received grant funding from Abbott, Boston Scientific, and Medtronic Canada, and consulting fees from Arca Biopharma (all unrelated to this work). The remaining authors declare that the research was conducted in the absence of any commercial or financial relationships that could be construed as a potential conflict of interest.

## Publisher’s Note

All claims expressed in this article are solely those of the authors and do not necessarily represent those of their affiliated organizations, or those of the publisher, the editors and the reviewers. Any product that may be evaluated in this article, or claim that may be made by its manufacturer, is not guaranteed or endorsed by the publisher.

## Abbreviations

AF: Atrial fibrillation
BSA: Body surface area
CIROC: Cardiovascular Imaging Registry of Calgary
CMR: Cardiovascular magnetic resonance imaging
DAD: Discharge Abstract Database
EF: Ejection fraction
GFR: Glomerular filtration rate
HV: Healthy volunteers
ICD-10, International Classification of Diseases: Tenth Revision
NACRS: National Ambulatory Care Reporting System
LAmax: Maximum left atrial volume
LAmin: Minimum left atrial volume
LApre-systole: Left atrial volume prior to LA systole
LV: Left ventricle
NCT: ClinicalTrials.gov Identifier
NYHA: New York Heart Association
PA: Pulmonary artery
PVI: Pulmonary vein isolation.
