# Supplementary material for: Influence of Sex-Based Differences in Cardiac Phenotype on Atrial Fibrillation Recurrence in Patients Undergoing Pulmonary Vein Isolation
Source: Front Cardiovasc Med. 2022 Jul 28;9:894592. doi: 10.3389/fcvm.2022.894592 (PMC9366168; doi:10.3389/fcvm.2022.894592)

SUPPLEMENTAL APPENDIX

Supplemental tables 1-6: pages 2-11

Supplemental figures 1 & 2: pages 12-13

Supplemental tables

Supplemental Table 1. Characteristics of healthy volunteers, stratified by sex

**Characteristic**	**Overall**<br><br>**N = 101**	**Males**<br><br>**N = 47 (47%)**	**Females**<br><br>**N = 54 (53%)**	**p-value**
**Non-imaging variables**				
Age	43.2 ± 14.6	45.1 ± 15.3	41.6 ± 13.9	0.24
**Body surface area (BSA, m^2)**	1.9 ± 0.2	2.0 ± 0.2	1.7 ± 0.2	**<0.001***
**Body Mass Index (BMI, kg/m^2)**	25.2 ± 3.7	26.3 ± 3.3	24.2 ± 3.8	**0.004***
Heart rate, bpm	63.0 (56.0, 70.0)	61.0 (54.5, 67.5)	64.5 (59.0, 70.0)	**0.018***
Systolic blood pressure, mmHg	112.7 ± 12.3	115.2 ± 11.8	110.7 ± 12.5	0.077
Diastolic blood pressure, mmHg	66.5 ± 10.0	67.7 ± 11.0	65.5 ± 9.1	0.31
**CMR variables**				
**LV EDV, ml/m^2**	81.2 ± 12.3	84.9 ± 11.8	78.1 ± 12.0	**0.005***
**LV ESV, ml/m^2**	29.5 ± 6.5	31.6 ± 5.9	27.6 ± 6.4	**0.002***
LV EF, %	63.9 ± 4.5	62.9 ± 3.7	64.8 ± 5.0	**0.030***
**LV mass, g/m^2**	48.7 ± 12.2	57.2 ± 11.8	41.4 ± 6.6	**<0.001***
**RV EDV, ml/m^2**	89.2 ± 14.4	96.1 ± 12.9	83.1 ± 12.9	**<0.001***
**RV ESV, ml/m^2**	38.2 ± 8.9	42.8 ± 7.9	34.3 ± 7.9	**<0.001***
RV EF, %	57.4 ± 5.1	55.6 ± 4.4	59.0 ± 5.1	**<0.001***
LAmax, ml	66.5 (54.8, 79.0)	73.4 (64.0, 89.3)	61.4 (52.6, 70.3)	**<0.001**
**LAmax, ml/m^2**	34.9 (31.1, 42.4)	34.8 (31.5, 44.4)	34.9 (31.1, 40.2)	0.47
LAmin, ml	25.3 (19.8, 29.7)	28.8 (23.3, 38.3)	21.5 (19.0, 25.8)	**<0.001***
**LAmin, ml/m^2**	13.7 (11.3, 16.1)	14.6 (11.4, 18.2)	13.0 (11.3, 14.9)	0.12
LApre-systole, ml	43.9 (34.9, 52.2)	52.0 (42.4, 62.8)	36.8 (33.4, 47.5)	**<0.001***
**LApre-systole, ml/m^2**	23.5 (20.0, 28.2)	24.4 (20.5, 30.6)	22.2 (19.5, 26.6)	0.073
LA global EF, %	62.4 (56.9, 67.0)	60.0 (55.3, 65.6)	63.5 (57.8, 67.4)	0.082
LA booster EF, %	42.2 (39.0, 46.2)	42.2 (38.0, 46.0)	42.5 (39.9, 46.1)	0.94
LA conduit EF, %	33.9 (25.4, 41.0)	30.5 (25.2, 39.5)	35.1 (28.2, 42.2)	0.065

Characteristic	Overall<br><br>N = 101	Males<br><br>N = 47 (47%)	Females<br><br>N = 54 (53%)	p-value

Values are mean ± SD or median (Q1, Q3). BSA indicates body surface area; BMI, body mass index; LV, left ventricle; EDV, end-diastolic volume; ESV, end-systolic volume; EF, ejection fraction; RV, right ventricle; LA, left atrium; LAmax, maximum LA volume; LAmin, minimum LA volume; and LApre-systole, LA volume pre-atrial systole.
*p<0.05.

Supplemental Table 2. Baseline CMR measurements of right atrial volumes and pulmonary venous and arterial measurements (non-Z-scores) for overall cohort, stratified by sex

Characteristic	Overall<br>N = 204	Males<br>N = 153 (75%)	Females<br>N = 51 (25%)	p-value
**Right atrium**				
RAmax, ml	95.6 (78.0, 116.3)	99.5 (82.9, 118.6)	77.3 (56.7, 96.1)	**<0.001***
**RAmax, ml/m^2**	45.0 (36.2, 56.5)	45.5 (38.7, 55.4)	41.8 (30.5, 56.6)	**0.024***
RAmin, ml	52.4 (36.6, 67.0)	54.9 (40.2, 70.6)	38.6 (28.1, 47.5)	**<0.001***
**RAmin, ml/m^2**	24.7 (17.5, 31.7)	25.6 (18.1, 32.8)	20.2 (13.7, 27.2)	**0.008***
**Pulmonary veins (3D-MRA)**				
**LIPV area, cm^2**	6.8 (5.3, 7.9)	6.9 (5.3, 7.9)	6.2 (5.1, 8.0)	0.37
**LSPV area, cm^2**	8.3 (6.8, 10.3)	8.5 (6.8, 10.6)	7.9 (6.2, 9.4)	0.11
**RIPV area, cm^2**	9.5 (7.5, 11.7)	9.6 (7.8, 11.9)	9.4 (7.4, 10.9)	0.12
**RSPV area, cm^2**	10.0 (8.2, 12.8)	10.2 (8.5, 13.1)	9.5 (7.4, 11.5)	**0.034***
Left common trunk present	31 (15%)	22 (14%)	9 (18%)	0.57
Separate right middle vein present	36 (18%)	28 (18%)	8 (16%)	0.67
**Pulmonary Arteries (3D-MRA)**				
**MPA area, cm^2**	24.6 ± 6.2	24.9 ± 6.4	23.6 ± 5.5	0.16
**RPA area, cm^2**	15.5 ± 4.5	16.0 ± 4.5	14.2 ± 4.3	**0.015***
**LPA area, cm^2**	16.0 ± 3.6	16.3 ± 3.5	15.3 ± 4.0	0.12

Values are mean ± SD, median (Q1, Q3), or number (%). RA indicates right atrium; RAmax, maximum RA volume; RAmin, minimum RA volume; 3D-MRA, three-dimensional magnetic resonance angiography; LIPV, left inferior pulmonary vein; LSPV, left superior pulmonary vein; RIPV, right inferior pulmonary vein; RSPV, right superior pulmonary vein; MPA, main pulmonary artery; RPA, right pulmonary artery; and LPA, left pulmonary artery.
*p<0.05

Supplemental Table 3. Univariable associations of CMR right atrial volumes and pulmonary venous and arterial parameters with the primary clinical outcome in the overall cohort

**Characteristic***	**HR (95% CI)†**	**p-value**
**Right atrium**		
**RAmax, per 1 ml/m²**	1.002 (0.989-1.015)	0.81
**RAmin, per 1 ml/m²**	1.005 (0.989-1.021)	0.55
**Pulmonary veins**		
**LIPV area, per 1 cm²**	1.044 (0.957-1.138)	0.33
**LSPV area, per 1 cm²**	1.039 (0.957-1.128)	0.36
**RIPV area, per 1 cm²**	1.028 (0.971-1.09)	0.34
**RSPV area, per 1 cm²**	1.044 (0.988-1.103)	0.12
Left common trunk present	0.821 (0.448-1.506)	0.52
Separate right middle vein present	0.820 (0.471-1.426)	0.48
**Pulmonary arteries**		
**MPA area, per 1 cm²**	1.022 (0.989-1.055)	0.20
**RPA area, per 1 cm²**	0.991 (0.946-1.038)	0.69
**LPA area, per 1 cm²**	1.004 (0.948-1.064)	0.88

HR indicates hazard ratio; CI, confidence interval; RAmax, maximum RA volume; RAmin, minimum RA volume; LIPV, left inferior pulmonary vein; LSPV, left superior pulmonary vein; RIPV, right inferior pulmonary vein; RSPV, right superior pulmonary vein; MPA, main pulmonary artery; RPA, right pulmonary artery; LPA, left pulmonary artery.
*All numerical variables are non-Z-score values
†Calculated per 1 unit increase for all numerical variables

Supplemental Table 4. Univariable associations of non-imaging variables with the primary clinical outcome in the overall cohort (both sexes) after excluding the six patients for whom the outcome of AF recurrence was solely based on ICD-10 coding

Characteristic	HR (95% CI)	p-value
Age, per 1 year	1 (0.977-1.025)	0.98
Female sex	1.620 (1.034-2.545)	**0.035***
Dyspnea (NYHA class II-IV)	1.090 (0.6978-1.699)	0.71
Diabetes mellitus	0.576 (0.182-1.822)	0.35
Hypertension	1.110 (0.704-1.764)	0.64
Hyperlipidemia	0.820 (0.525-1.280)	0.38
Hypothyroidism	1.470 (0.840-2.562)	0.18
Hyperthyroidism	0.777 (0.191-3.159)	0.73
Chronic kidney disease	1.550 (0.799-2.991)	0.20
Smoking		
Never	Reference category	
Current	0.952 (0.474-1.912)	0.89
Former	0.985 (0.490-1.979)	0.97
Regular alcohol consumption (at least 1 drink/day)	0.610 (0.338-1.101)	0.10
Caffeine consumption		
None	Reference category	
Occasional (<1 drink/day)	0.638 (0.260-1.563)	0.33
Regular (at least 1 drink/day)	0.636 (0.272-1.489)	0.30
QoL (rating on 0-100 scale)	0.997 (0.985-1.010)	0.69
Baseline medications		
Aspirin	1.102 (0.606-2.052)	0.73
Beta-blocker	1.020 (0.654-1.596)	0.93
ACEi/ARB	1.060 (0.681-1.663)	0.78
Calcium channel blocker	1.400 (0.872-2.236)	0.16
Anti-coagulant	1.090 (0.473-2.492)	0.85

Characteristic	HR (95% CI)	p-value
Anti-arrhythmic	0.580 (0.375-0.898)	**0.015***
Digoxin	0.476 (0.150-1.507)	0.21
Loop diuretic	1.390 (0.756-2.559)	0.29
Lipid-lowering	0.806 (0.511-1.269)	0.35
Body surface area, per m^2	0.658 (0.266-1.632)	0.37
BMI, per 1 kg/m^2	0.989 (0.945-1.034)	0.62
Obesity (BMI ≥30 kg/m^2)	0.935 (0.593-1.473)	0.77
Heart rate, per 1 bpm	1.010 (0.998-1.018)	0.11
Systolic blood pressure, per 1 mmHg	1.010 (0.996-1.027)	0.15
Diastolic blood pressure, per 1 mmHg	1.010 (0.987-1.033)	0.42
Atrial fibrillation type – non-paroxysmal (vs paroxysmal)	1.260 (0.816-1.941)	0.30
Labs		
Haemoglobin, per 1 g/L	0.993 (0.978-1.009)	0.38
GFR, per 1 mL/min/1.73 m^2	1.000 (0.992-1.008)	0.997
Ablation procedure		
Sinus rhythm at onset of procedure	0.723 (0.473-1.107)	0.18
Ablation count	0.998 (0.990-1.007)	0.67
Total ablation time, per 1 s	1 (1-1)	0.53
Maximum power, per 1 watt	0.997 (0.928-1.070)	0.92
Left atrial roof line	2.130 (1.029-4.420)	**0.042***
Posterior isolation/posterior box	1.610 (0.397-6.570)	0.50
PVAC catheter used	1.070 (0.262-4.338)	0.93
3D mapping system used	0.999 (0.139-7.183)	0.999
Complete pulmonary vein isolation achieved	0.824 (0.334-2.036)	0.68

HR indicates hazard ratio; CI, confidence interval; NYHA, New York Heart Association; CKD, chronic kidney disease; QoL, quality of life; ACEi, angiotensin converting enzyme inhibitor; ARB, angiotensin receptor blocker; BMI, body mass index; and GFR, glomerular filtration rate.
*$p<0.05$

Supplemental Table 5. Univariable associations of Z-score values for CMR chamber volumes and ejection fraction with the primary clinical outcome in the overall cohort (both sexes) following the exclusion of six patients for whom the outcome of AF recurrence was solely based on ICD-10 coding.

CMR variables	HR (95% CI)*	p-value
LV EDV	1.100 (0.918-1.312)	0.31
LV ESV	1.040 (0.913-1.189)	0.54
LV EF	1.010 (0.921-1.107)	0.83
LV mass	1.130 (0.934-1.377)	0.21
RV EDV	1.070 (0.911-1.253)	0.42
RV ESV	1.060 (0.891-1.257)	0.52
RV EF	1.010 (0.890-1.147)	0.87
LAmax†	1.040 (0.872-1.240)	0.52
LAmin†	**1.084 (0.955-1.229)**	**0.001‡**
LApre-systole†	**1.168 (1.033-1.321)**	**0.045‡**
LA global EF†	0.884 (0.673-1.162)	0.11
LA booster EF†	**0.992 (0.768-1.282)**	**0.017‡**
LA conduit EF†	0.937 (0.650-1.350)	0.67

HR indicates hazard ratio; CI, confidence interval; LV, left ventricle; EDV, end-diastolic volume; ESV, end-systolic volume; EF, ejection fraction; RV, right ventricle; LA, left atrium; LAmax, maximum LA volume; LAmin, minimum LA volume; and LApre-systole, LA volume pre-atrial systole

*Calculated per 1 unit increase in Z-score for all variables, except for restricted cubic spline (RCS) transformed ones calculated for a Z-score of 2.0 versus 1.0

†RCS transformation used, with HR and 95% CI calculated for a Z-score of 2.0 versus 1.0

‡p<0.05

Supplemental Table 6. Multivariable models for associations of Z-scores for left atrial CMR parameters with the primary clinical outcome in overall cohort (both sexes) after excluding the six patients for whom the outcome of AF recurrence was solely based on ICD-10 coding.

Parameter	HR (95% CI)*	p-value
**Model 1. LAmin**		
Age, per 1 year	0.987 (0.962-1.013)	0.34
Diabetes	0.545 (0.163-1.816)	0.32
Hypertension	1.373 (0.818-2.306)	0.23
Regular alcohol consumption (at least 1 drink/day)	0.523 (0.274-1.001)	0.050
Pre-procedural anti-arrhythmic medication	**0.485 (0.298-0.791)**	**0.004†**
LAmin‡	**1.110 (0.972-1.269)**	**<0.001†**
**Model 2. LApre-systole**		
Age, per 1 year	0.990 (0.964-1.016)	0.46
Diabetes	0.598 (0.181-1.974)	0.40
Hypertension	1.338 (0.802-2.234)	0.27
Regular alcohol consumption (at least 1 drink/day)	0.538 (0.282-1.025)	0.060
Pre-procedural anti-arrhythmic medication	**0.500 (0.303-0.826)**	**0.007†**
LApre-systole‡	**1.210 (1.068-1.370)**	**0.011†**
**Model 3. LA booster EF**		
Age, per 1 year	1.000 (0.975-1.026)	0.99
Diabetes	0.619 (0.187-2.046)	0.43
Hypertension	1.444 (0.846-2.464)	0.18
Regular alcohol consumption (at least 1 drink/day)	**0.524 (0.275-0.999)**	**0.0496†**
Pre-procedural anti-arrhythmic medication	**0.494 (0.304-0.804)**	**0.005†**
LA booster EF‡	**0.897 (0.686-1.175)**	**0.011†**

HR indicates hazard ratio; CI, confidence interval; LA, left atrium; LAmin, minimum LA volume; and LApre-systole, LA volume pre-atrial systole; LApre-systole, LA volume pre-atrial systole; and EF, ejection fraction.
*Calculated per 1 unit increase for all numerical variables, except for restricted cubic spline (RCS) transformed ones calculated for a Z-score of 2.0 versus 1.0
†$p<0.05$
‡ RCS transformation used, with HR and 95% CI calculated for a Z-score of 2.0 versus 1.0

Supplemental Figure 1. Kaplan-Meier curves for freedom from recurrence of atrial fibrillation for females versus males after excluding the six patients for whom the outcome of AF recurrence was solely based on ICD-10 coding.

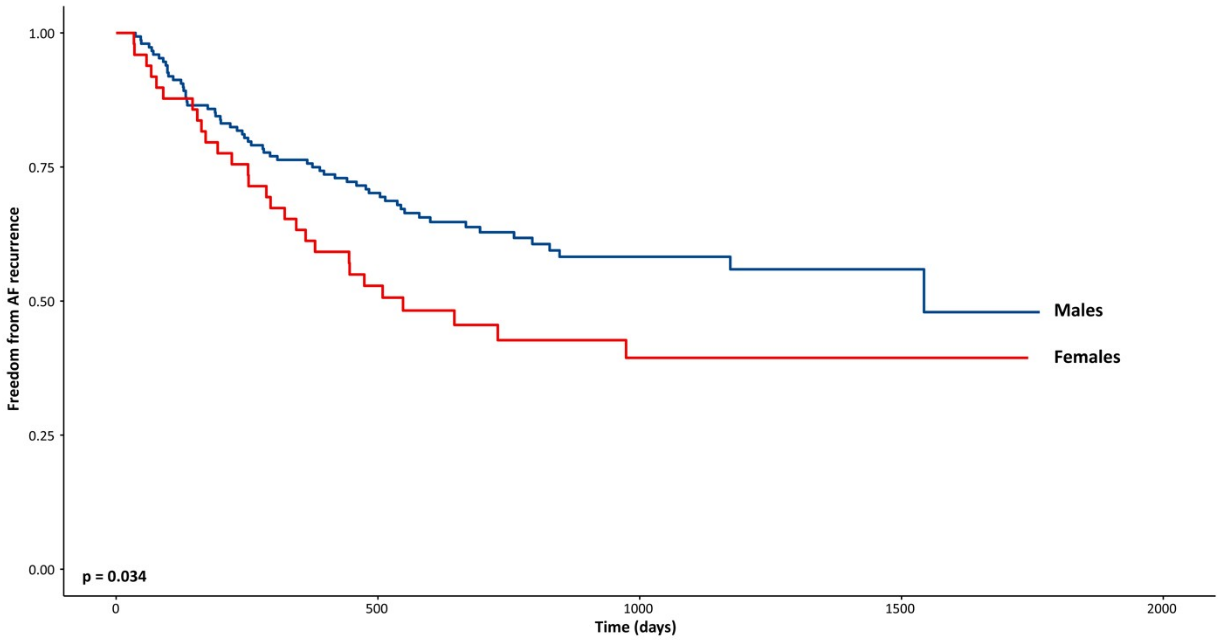

Number at risk

	0	500	1000	1500	2000
Males	149	96	36	7	0
Females	49	24	11	3	0

Supplemental Figure 2. Kaplan-Meier curves for left atrial (LA) parameters as predictors of AF recurrence after excluding the six patients for whom the outcome of AF recurrence was solely based on ICD-10 coding. Unadjusted Kaplan-Meier curves describe associations between lower versus higher Z-scores of LA booster ejection fraction (EF; panel A), minimum LA volume (LAmin; panel B), and pre-systole LA volume (LApre-systole; panel C) values and freedom from atrial fibrillation recurrence following pulmonary vein ablation.

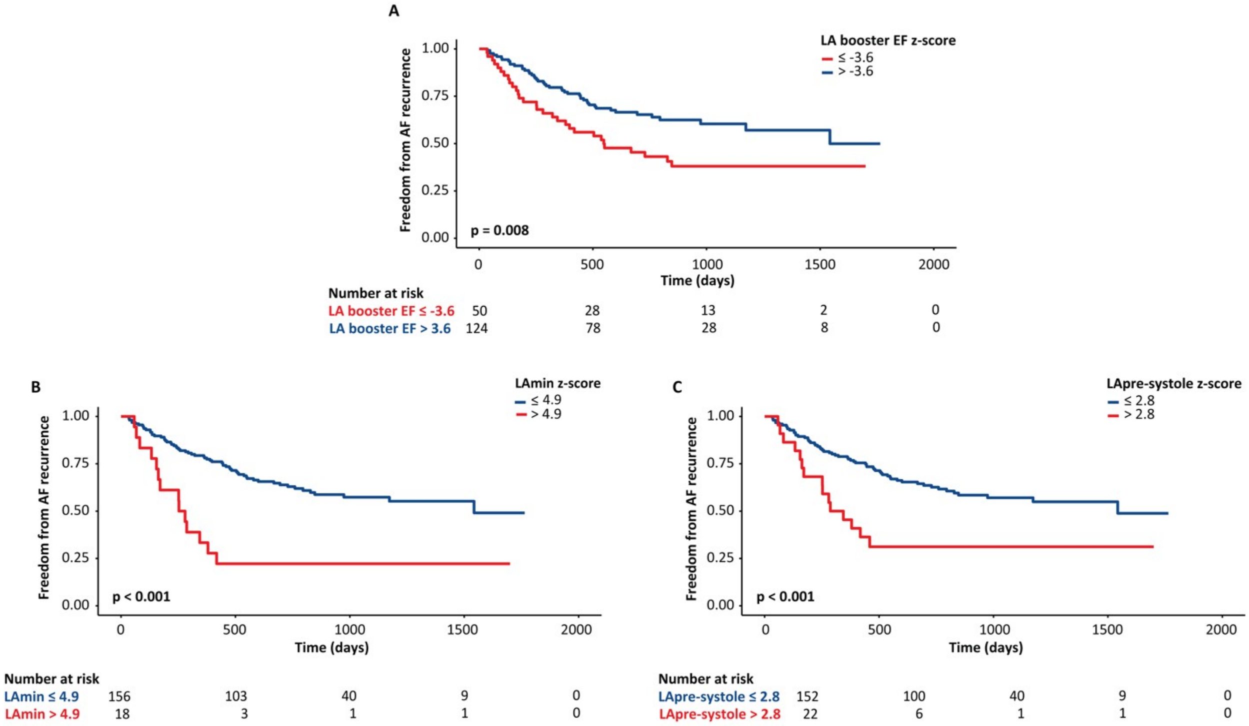

Supplement: Supplementary file 1 [file Data_Sheet_1.pdf]
